# Randomized Controlled Evaluation of a Group-Based Training for Parents of Adolescents with Gaming Disorder or Social Network Use Disorder

**DOI:** 10.3390/ijerph20010272

**Published:** 2022-12-24

**Authors:** Isabel Brandhorst, Patrizia Lahres, Sara Hanke, Anil Batra, Tobias Renner, Gottfried Barth, Katajun Lindenberg, Eva Vonderlin, Kay Petersen

**Affiliations:** 1 Department of Psychiatry, Psychosomatics and Psychotherapy in Childhood and Adolescence, University Hospital Tuebingen, 72076 Tubingen, Germany; 2 Section of Addiction Medicine and Addiction Research, Department of Psychiatry and Psychotherapy, University Hospital Tuebingen, 72076 Tuebingen, Germany; 3 Child and Adolescent Psychotherapy, Institute for Psychology, Goethe-University Frankfurt, 60486 Frankfurt, Germany; 4 Child and Adolescent Psychotherapy, Institute for Psychology, University of Heidelberg, 69115 Heidelberg, Germany

**Keywords:** gaming disorder, social network use disorder, internet use disorder, parents, family, group training, intervention

## Abstract

Background: Internet Use Disorder (IUD), Gaming Disorder (GD), and Social Network Use Disorder (SNUD) are common phenomena among adolescents and young adults. Negative consequences of such disorders can be observed in the individuals themselves and in the family system. Although parents can influence their children in many ways, they are rarely considered in interventions. The present study examines the effectiveness of a group-based training for parents of adolescents with IUD, GD, or SNUD. Methods: A total of 76 parents of adolescents (12 to 20 years) were randomly assigned to the intervention group (IG) or the waiting list control group. Parents in the IG participated over eight weeks in six sessions of training (topics: psychoeducation, parenting behaviour, parent-child relationships, parent-child communication, and stress and relaxation). Questionnaires on adolescent symptomatology, parent-child relationships, and parental burden were collected before and after the intervention/waiting period. Results: The training reduced the IUD symptomatology of adolescents from the parents’ perspective. GD symptomatology improved for at-risk users, though not for pathological users. Some aspects of the parent-child relationships improved in the mothers’ judgment. Parental stress was already low before the training. Conclusions: The presented parent group training can be used to improve IUD symptomatology in adolescents and is effective in the context of early intervention for at-risk computer gamers.

## 1. Introduction

For most young people, computer games or social networks are an enjoyable pastime that provides pleasure and social networking [1,2]. However, some people fail to manage these digital opportunities in a controlled manner and develop an Internet Use Disorder (IUD), or more specifically a Gaming Disorder (GD) or Social Network Use Disorder (SNUD). According to ICD-11, GD occurs when there is a loss of control over Internet use and the use becomes an increasingly higher priority in life, resulting in negative consequences. The criteria also require that these symptoms persist for more than 12 months and result in a meaningful impairment in personal, educational/vocational, and/or social life [3]. 

The international prevalence of IUD is estimated at 7.02% [4]. In Germany, current estimates among adolescents indicate a prevalence of GD of 3.5%, of SNUD of 2.6%, and of the combination of both of around 0.5% [5]. The prevalence rates of IUD increase during adolescence [6]. The impacts of such a disorder can be multifaceted. Regarding GD, associations with depressive symptoms, withdrawn behaviour, anxiety, compulsions, social problems, ADHD, higher stress levels, a lower quality of life, lower interpersonal trust, and lower familial functioning are described [5,7,8]. In the context of SNUD, mental distress, a negative self-image, self-harming behaviour, depression, suicidal ideation, and sleep deprivation, as well as negative effects on cognitive control, academic performance, and socioemotional functioning have been reported [2,5]. 

A wide variety of interventions, such as group therapy, sports interventions, and Internet-, mindfulness-, or medication-based interventions are available for treatment, with cognitive behavioural therapy proving superior [9]. Interventions that intensively involve the parents or the family system are rare. A recent meta-analysis shows that, of 59 published intervention studies, only five involved the family system [9]. Yet there are many good reasons to address parents or the family system. 

First, the parent-child relationship is one of the most influential family factors for the symptomatology of affected individuals [10,11]. This relationship is not only observed in younger children but can also be found in young adults [11]. Long-term studies confirm that impairments in the parent-child relationship may precede a later development of IUD problems [12]. The influence of the mother-child or father-child relationship is often valued differently, but the data remain heterogeneous [10,13]. One aspect of the parent-child relationship is parental warmth, which has been shown in meta-analyses to be negatively associated with IUD problems in children [14]. Parent-child communication can be a way to express emotional warmth toward the child. One study showed that problems in the quality of parent-child interaction are associated with a later development of IUD symptomatology. The study also showed that the quantity of communication is more likely to be impaired as a result of symptomatology [15]. Several cross-sectional studies describe more frequent conflict and negative communication in the families of children with IUD or GD [16,17]. Changing parent-child communication to focus more on the expression of emotional needs and the communication of emotional warmth is therefore a promising approach to intervention. 

Furthermore, parents should be addressed when treating their children, because they can make an impact through their parenting and educational strategies [18]. At the same time, it is unclear what effects media-specific parenting strategies can have [14]. A recent meta-analysis found a positive association between restrictive parental media education and IUD symptoms in older adolescents, whereas this association did not exist in children [14]. Other studies have shown that inconsistent, chaotic, or rigid rules were associated with more IUD problems [18,19,20]. There is a lack of studies designed to identify causal relationships. Interventions should target consistent, clear, and flexible media use rules in families and be cautious about recommendations to limit media use times among youth. 

Parents should be integrated into the treatment of children and adolescents with INS, or even deserve a separate counselling focus, as they are co-burdened to a serious degree [21]. For example, associations have been reported between IUD in children and depression or anxiety in parents [22,23], but the findings are heterogeneous and the causality is unclear. In addition, associations exist between IUD symptoms in parents and in their children [24]. Treatment programs should consider the possible increased psychological burden placed on parents, their heightened stress levels, and their function as parental role models. 

There are individual programs that involve parents in their children’s treatment. Liu et al. achieved reductions in IUD symptoms with their multifamily therapy (the topics included family communication and parent-child relationships) [25]. Han et al. reduced screen time for those with IUD by increasing family time, among other things [26]. Psychoeducational elements for parents accompanying a GD intervention for adolescents did not lead to better treatment outcomes in one study, but they did lead to lower dropout rates [27]. Li et al. examined a parent-only program to prevent GD symptomatology in high school students (content: parental monitoring, parental care, and psychoeducation) [28]. They observed a reduction in screen time, reduced use of aggressive Internet content, and a reduction in GD symptoms compared with the control group. To date, there is no evidence of efficacy for interventions aimed exclusively at parents of adolescents with IUD, GD, or SNUD. The present study aims to fill this gap.

For this reason, the ISES! Group Training (“Internet Addiction: Empowering Parents!”/original: “Internetsucht: Eltern staerken!”) for parents of adolescents and young adults with GD or SNUD was developed in Tuebingen. The manualized group training with its six sessions is aimed exclusively at parents and includes the topics of psychoeducation, parenting behaviour, parent-child relationships, parent-child communication, and stress and relaxation for parents. The results of a pilot study showed that participants rated the training well, children’s computer game behaviour improved from the parents’ perspective, psychological abnormalities were reduced, and children’s quality of life increased [29]. Furthermore, an online training was developed, which is currently under evaluation and will not be presented further here [30].

The present study describes the results of a multicentre randomized controlled efficacy trial of the ISES! Group Training. The following hypotheses are formulated: Training decreases adolescents’ IUD, GD, or SNUD symptomatology and parents’ distress, increases adolescents’ readiness for treatment, and improves parent-child relationships.

## 2. Materials and Methods

### 2.1. Design and Procedure

The study was conducted from July 2021 to July 2022 as a cooperation effort of the Department of Child and Adolescent Psychiatry and the Department of Adult Psychiatry, Section of Addiction Medicine and Addiction Research at the University Hospital Tuebingen. Other recruitment centres were the University Outpatient Clinic of the University of Heidelberg and the Behaviour Therapy Outpatient Clinic of the University of Frankfurt. A positive vote of the Ethics Committee of the Medical Faculty of Tuebingen was available (registration number: 916/2020BO1, date of approval 30 May 2021). 

Parents of adolescents and young adults were recruited via newspaper articles, a project website, and therapists. Families (single parents or pairs of parents) were alternately assigned to the intervention group (IG) or the waiting list control group (CG) upon receipt of informed consent (quasi-random sampling). Both groups participated in a digital pseudonymized pre- and post-survey via the provider SoSci Survey at eight-week intervals (www.soscisurvey.de, accessed on 31 October 2022). The IG received the eight-week intervention right after the pre-survey. The CG received no specific intervention between the pre- and post-surveys and participated in the training after completing the post-survey. Follow-up surveys took place six and twelve months after the last training appointment for both groups. The follow-up surveys have not been completed at the current time and are therefore not reported here.

The inclusion criteria for participation in the study were the informed consent of the parents, adolescents between 12 and 21 years of age, and IUD, GD, or SNUD symptomatology assessed subjectively and globally by the parents (no query of specific criteria). Adolescents were also required to live mainly with the participating parent, and parents were required to speak sufficient German. 

Adolescents could also optionally participate in a pre- and post-survey before the waiting period/parent intervention. This optional survey of the youth took place online, and the request for the survey came via email. There was no contact between the study staff and the adolescents at any time and no adolescent participation in the intervention. 

### 2.2. Assessments

Social Data: Baseline sociodemographic data for both parents and child were collected (age, gender, highest education, cultural affiliation, school of the child, school attendance).

Compulsive Internet Use Scale—Parent Version (CIUS-P): The CIUS [31] (translated by K. Petersen) is the most widely used scale for the assessment of Internet-related disorders in Germany. The new parent version, CIUS-P, has been developed at the Department of Psychiatry, Psychosomatics and Psychotherapy in Childhood and Adolescence at the University Hospital in Tuebingen and is currently being psychometrically tested. The internal consistency of the questionnaire in our population was between 0.82 and 0.84 (Cronbachs Alpha). 

Video Game Dependency Scale—Parental Version (CSAS-FE): The CSAS [32] is a questionnaire to assess GD according to the nine DSM-5 criteria. The questionnaire allows a suspicion-diagnostic classification of at-risk users (2–4 fulfilled criteria) and pathological users (5–9 criteria). The CSAS is probably the best studied questionnaire for GD in Germany and shows good values regarding reliability and validity. We used the parent version (CSAS-FE, 18 items), which is not validated in German, but which had already proven to be sensitive to change in the pilot study [29].

Single questions regarding symptomatology and readiness for treatment: Individual questions were asked about symptom severity, motivation, and the COVID-19 pandemic. The questions in detail were: (1) Global rating, parent’s view: “How problematic do you think your child’s Internet use behaviour is?” (1 = not problematic, 10 = very problematic). (2) Global rating, adolescent’s’ view: “How problematic does your child think his or her own Internet use behaviour is?” (1 = not problematic, 10 = very problematic). (3) Importance of help: “How important do you think it would be for your child to seek professional help to change the problem behaviour?” (1 = not important, 10 = very important). (4) Motivation to seek help: “How high do you rate your child’s motivation to seek professional help to change the problem behaviour?” (1 = low, 10 = very high). (5) Motivation to talk: “How high do you rate your child’s current motivation to talk to you about his or her problem behaviour?” (1 = low, 10 = very high). (6) Motivation to change: “How high do you estimate your child’s current motivation is to change his or her problem behaviour?” (1 = low, 10 = very high). (7) COVID-19 pandemic: “Do you feel that the Corona lockdown has changed your child’s Internet use?” (“The use became even more problematic”/“The use became problematic for the first time”/“The use became less problematic”/“The lockdown has not changed the situation”).

Parent-Child Inventory (EKI): Since no already standardized questionnaires in the German language were found that fit our target group, the EKI was developed by K. Petersen. It is designed to capture the parent-child relationship on three scales (caring, empathy, congruence) as a description of the adolescent from the parent’s perspective. The internal consistency of the questionnaire in our population was between 0.71 and 0.87 (Cronbachs Alpha).

Parental Representation Screening Questionnaire—Parental Version (PRSQ-P): The PRSQ [33] in its original version captures the representation of the parent-child relationship from the child’s perspective on three resource scales (cohesion, identification, autonomy), five risk scales (conflicts, rejection/neglect, punishment, emotional burden, fears/overprotection), and one additional scale aid. For the present study, we used an adapted version from the parents’ perspective, which has not yet been published. The internal consistency of the questionnaire in our population was between 0.59 and 0.72 for the resource scales, between 0.28 and 0.82 for the risk scales, and between 0.60 and 0.74 for the scale aid (Cronbachs Alpha).

Depression Anxiety Stress Scales (DASS): The DASS [34,35] is an established screening instrument for mental distress with the subscales depression (Cut-off > 10), anxiety (Cut-off > 6), and stress (Cut-off > 10). The internal consistency of the scales is 0.88 for the depression scale, 0.76 for the anxiety scale, and 0.86 for the stress scale [34].

Single questions regarding conflicts and burdens on the parents: Individual questions were asked about family conflicts and parental burden. The questions in detail were: (1) “How often did aggressive physical altercations with your child occur in connection with disputes about media use?” (1 = never, 5 = very often). (2) “How often does media use cause arguments in the family?” (less than weekly/about once a week/several times a week/daily/several times a day). (3) “How often do you feel burdened by your child’s Internet use?” (1 = never, 5 = very often). (4) “How much do you feel burdened by your child’s Internet use?” (lightly burdened/moderately burdened/severely burdened).

### 2.3. Intervention

The on-site ISES! Group Training took place in six sessions of 90 minutes each spread over eight weeks (session 1–4 weekly, session 5 + 6 every 2 weeks). Four to seven families with one or two parents each could participate in a training group (min. 4, max. 14 persons per group). The content of the training is described in Table 1 and includes the following topics: psychoeducation, parenting behaviour, parent-child relationships, parent-child communication, stress and relaxation for parents. Each session included an exchange between the group participants, exercises in the group (e.g., working together on the flipchart, written exercises), exercises to do at home, and content taught by group trainers. The trainers were therapeutically trained (e.g., psychotherapists in advanced training) and had experience in leading a parent group. Standardized implementation of the training content was ensured by a trainer manual. Participants also received a detailed handout. Trainers from external study centres were trained in a three-hour session prior to the start of the group training.

### 2.4. Sample Calculation and Description

An analysis with the program G*Power [36], carried out before the start of the study, aimed at a total sample size of N = 30 families with 42 participants and 4 groups in order to identify existing between-group effects between the pre- and post-measurement time points in a 2 × 2 factorial MANOVA (calculation basis: effect size from pilot study d = 0.53; alpha 0.05; power 0.8, number of groups 2; measurement time points 2). 

A total of 76 parents registered for the study. Fifty-nine parents completed the pre- and post-diagnostic surveys and were included in the analysis (IG N = 33, CG N = 26). All families in the IG participated in at least 4 training sessions; therefore, there was no dropout during training participation (Tuebingen N = 39, Frankfurt N = 11, Heidelberg N = 9).

### 2.5. Statistical Evaluation

The statistical program IBM SPSS Statistics 27 was used for data evaluation. Questionnaires from individual persons were excluded from the analysis if more than 33% of the answers were missing. In the case of fewer missing values, they were replaced by the mean value of the questionnaire of the person concerned. Outliers conspicuous in the frequency analysis led to the exclusion of the respective questionnaire if the value deviated from the mean by more than two standard deviations (rounded to whole numbers). 

The change in mean values in the two groups over the two measurement time points was treated multivariately for interval scales in an analysis of variance with repeated measures. The Pillai trace test was evaluated. For non-interval scales, the Wilcoxon signed-rank test was used, and changes in the groups over time were considered separately. Pre-group differences were tested using *t*-tests. Nonparametric test procedures were used for violations of the normal distribution, as measured by the Kolmogorov-Smirnov test, or nominal or ordinal scales. Changes in the participant-reported frequency of family disputes were assessed using the Wilcoxon signed-rank test.

## 3. Results

### 3.1. Sample Description

The 59 individuals who participated in the training (M = 50.26 years, SD = 4.31, Range = 40–61) were parents of 44 children (13.63% female; M = 14.68 years, SD = 2.10, Range = 12–20). The sociodemographic data for the parents and adolescents as well as information on Internet use from the first measurement time point can be found in Table 2. Fifteen parents (25.4%) stated at the first measurement point that they were in contact with a psychologist or psychiatrist regarding the media problem, 13.6% (N = 8) received other help (e.g., from the youth welfare office), and 61.0% (N = 36) had no support in this regard.

### 3.2. Symptomatology and Readiness for Treatment 

A total of 40.7% (N = 24) of the adolescents met the criteria for a gaming disorder (5–9 DSM-5 criteria) according to the CSAS-FE at the pre-measurement, while 30.5% (N = 18) were classified as at-risk users (2–4 criteria). Furthermore, 71.2% (N = 42) of parents reported that media use problems worsened during the COVID-19 pandemic, 23.7% (N = 14) reported a first-time occurrence of problems, 5.1% (N = 3) reported no change, and 0% reported improvement. Parents globally ranked their children’s media use as highly problematic during pre-measurement (M = 8.86, SD = 1.20; scale 1 = not problematic/10 = very problematic). From the parents’ perspective, their child would rate their own media use as significantly less problematic (M = 3.53, SD = 2.25; scale 1 = not problematic/10 = very problematic). At the pre-measurement point, the parents gave high scores on the single question regarding the importance of help (M = 8.22, SD = 2.28; scale 1 = not important/10 = very important).

The results of the pre-post analysis of the child’s symptomatology and readiness for treatment can be found in Table 3. A comparison of the IG and CG across the pre- and post-surveys showed a significant interaction effect for the CIUS-P. This effect was not found in the overall CSAS-FE score. An analysis of the diagnostic subgroups of the CSAS-FE revealed a significant interaction effect for at-risk users, but no effects for pathological users or users who met a maximum of one criterion. Significant interaction effects were found for the questions of whether the children were motivated, from the parents’ perspective, to engage with the parents about the problem behaviour or to change the problem behaviour. No effects were found for the question of whether the children were more willing to seek professional help.

### 3.3. Parent-Child Relationship

The results of the pre-post analysis of the parent-child relationship were evaluated both for parents together and for mothers and fathers separately. Table 4 shows the significant or almost significant results. All results of the PRSQ-P and EKI questionnaires can be found in Supplementary Table S1. The evaluation of the PRSQ-P revealed a significant interaction of the risk scale “emotional burden” for the parents together. No intervention effects were found for the other scales. An analysis of the mothers showed a significant interaction effect for the risk scale “emotional burden”. The changes in the resource scale “cohesion” and the neutral scale “aid” in the answers of the mothers were just shy of significance. The other scales showed no significant effects for the mothers. No significant effects were observed for the fathers in the PRSQ-P.

The evaluation of the EKI showed a tendency towards a significant interaction of the scale “child empathy” for the parents together. No intervention effects were found for the other scales. An analysis of the mothers showed a significant interaction effect of the scales “child authenticity” and a trending significant interaction for the scale “child empathy”. The other scales showed no significant effects for mothers. No significant effects were observed for fathers in the EKI.

### 3.4. Conflicts

At pre-measurement, 13.6% (N = 8) of the parents reported that aggressive physical confrontations with the child had occurred frequently or very frequently in relation to media use. An analysis of the pre-post data regarding the frequency of conflicts showed that the mean values of the IG were significantly reduced, while this change was not observed in the CG (IG: z = −3.01, *p* = 0.003; Pre: M = 3.79, Post: M = 3.00; CG: z = −0.73, *p* = 0.47; Pre: M = 3.19, Post: M = 3.08). Subgroup analysis showed that these changes in the IG were only observed in mothers and not in fathers (mothers: z = −2.75, *p* = 0.006; pre: M = 3.58, post: M = 2.63; fathers: z = −1.41; *p* = 0.159; pre: M = 4.07, post: M = 3.50). In addition, the frequency of conflicts in the IG reduced only among adolescents who mainly played computer games and not among adolescents who mainly used social media (Gaming: z = −2.87, *p* = 0.004; Pre: M = 4.13, Post: M = 2.80; Social Media: z = −1.39, *p* = 0.163; Pre: M = 3.43, Post: M = 2.71).

### 3.5. Parents’ Burden

The results of the DASS showed that, on average, both the mixed parent sample and mothers and fathers subgroups scored below the cut-offs of the questionnaire subscales (see Table 5). The change measure showed a significant increase in the depression subscale and in the DASS total value for fathers. No changes could be detected in either the subscales or the DASS total value for mothers. 

Comparing the mean scores in the stress subscale between parents of the diagnostic subgroups of the CSAS-FE revealed a significant difference between the parents of non- or inconspicuous users (M = 4.79, SD = 2.72) and at-risk users (M = 8.33, SD = 4.67; t(28.10) = −2.69, *p* = 0.012), and between the parents of pathological (M = 8.42, SD = 2.72) and non- or inconspicuous users (t(34.47) = 2.54, *p* = 0.016). There was no significant difference in the mean stress scores between parents of pathological and at-risk users (t(40) = −0.05, *p* = 0.961). 

At pre-measurement, parents reported feeling very frequently (59.3%/N = 35) and severely (64.4%/N = 38) burdened by their child’s media use. The pre-post comparison of IG and CG showed a significant interaction effect regarding the question of whether parents feel irritated by their child’s media use (F = 7.25, *p* = 0.009, effect size = 0.11; IG pre: M = 4.70, SD = 0.53, IG post: M = 4.12, SD = 0.93; CG pre: M = 4.62, SD = 0.70, CG post: M = 4.58, SD = 0.64).

## 4. Discussion

The present study describes the results of a multicentre randomized controlled efficacy trial of the ISES! Group Training. It examined whether the group training can reduce adolescents’ IUD, GD, or SNUD symptomatology from parents’ perspective, increase their readiness for treatment, improve parent-child relationships, and decrease parental burden.

### 4.1. Symptomatology and Readiness for Treatment

The results show that the ISES! Group Training led to reductions in adolescents’ IUD symptomatology from the parents’ perspective. Reductions in GD symptomatology were found for adolescents with at-risk use (2–4 DSM-5 criteria). Therefore, it can be concluded that the ISES! Group Training for IUD is effective. With respect to problematic computer game behaviour, the training leads to improvements mainly in the context of early intervention. This extends previous research showing that family therapy [25,26] and parent-only training can work in the context of prevention [27]. 

Looking at the CIUS-P mean scores in our sample (IG: M = 42, CG: M = 41), it is noticeable that the scores are very high compared to those from CIUS as a self-reporting instrument. In comparison, the psychometric evaluation of the CIUS for 14 to 17-year-old adolescents from the general population reported a cut-off value of 19 (75th percentile) [37]. A total of 99.93% of this norm sample reported scores below a score of 40, and another study described a critical threshold for pathological use of 28 [38]. Thus, the values of the CIUS as a self-reporting instrument found in the scientific literature are clearly lower than the values of the CIUS-P of our sample. On the one hand, these findings could be explained by the fact that parents tend to overestimate the IUD problem in their child, or that the children tend to underestimate their own problem. This would fit with the result of our sample that shows that parents globally rate their children’s media use as highly problematic, whereas parents believe that their children would rate their own behaviour as being significantly less problematic. However, experience with other GD questionnaires suggests that parents and children agree relatively well in their assessments (correlation of r = 0.78) [39]. Another interpretation could be that our sample is highly burdened. A psychometric evaluation of the CIUS-P comparing adolescent and parent data would be needed to conclusively assess this result.

Another goal was to examine whether the ISES! Group Training increases adolescents’ readiness for treatment. The results show that, according to parents’ judgment, adolescents in the IG have a higher willingness to change their problem behaviour after the training, while this willingness decreased in the CG. The adolescents’ readiness to seek professional help from the parents’ perspective was very low in both groups both before and after the training and did not change. The low willingness to seek treatment from the parents’ perspective allows for several interpretations. First, it could be that parents do not want (additional) professional help for their child. Already, 39% of the parents indicated that, prior to participation in the ISES! Group Training, they had had contact with professional help. On the other hand, it could be that the lack of problem insight is responsible for the low readiness for treatment and that the ISES! Group Training cannot change this insight or readiness, at least in the short term. In comparison, Bischof et al. [40] were able to demonstrate an improvement in treatment readiness in their training of the relatives of people with alcohol disorders. Follow-up analyses of the present data will clarify whether the readiness of this sample changes over time.

### 4.2. Parent-Child Relationships, Conflicts, and Communication

The results show positive developments in the parent-child relationships, but mostly only in the mothers’ judgment and partly not significantly. Mothers in the IG expressed improvements in family cohesion (expressing love, being there for each other, cuddling), child authenticity (child expressing thoughts and feelings, not pretending to play a role), child empathy (child’s interest in parent’s feelings and thoughts), and aid (child showing consideration, helping with chores) after the training. However, in some cases these results did not reach statistical significance, which might be attributed to the small sample size. In addition, the subscale “emotional burden” of the PRSQ-P increased (discussing worries together, asking the child for advice, comforting the parents). This scale actually belongs to the risk scales, as it is supposed to depict parentification that can be harmful to the development of a child. In our context, however, the scale can rather be interpreted positively as the child’s openness to the concerns of parents. The results suggest that the adolescents opened up more to the mothers as a result of the training, and more emotional warmth, empathy, and social support may have emerged. Since problems in the parent-child relationship and lack of emotional warmth are among the most relevant family influencing factors [12,14], it is of particular importance that the ISES! Group Training seem to be able to improve these aspects. Exciting questions for future analyses and studies include whether or not this development is also reflected in the children’s judgment, how these positive effects play out over time, and which interventions in the training triggered these developments. Furthermore, why fathers did not show these developments remains an open question. In the previous research literature, there are studies that assess the relationship with mothers as more relevant [41,42,43], as well as studies that emphasize the relationship with the father [41,44,45].

In 13.6% of families, physical conflicts between parents and child occurred frequently or very frequently because of media use. It remains unclear whether this percentage should be considered high or low. A recent study from China describes a prevalence of physical aggression among the adolescents in a population sample at 23.9% [46]. At the same time, this research showed a high correlation with IUD symptomatology. From practical experience with the ISES! Group Training, it can be reported that the physical conflicts between parent and child were associated with attempts by parents to enforce media-related regulations. Law et al. [47] reported in their study that open communication with parents about children’s media use was associated with lower media-related aggression potential among adolescents. These findings confirm the relevance of the communication and de-escalation aspects in the ISES! Group Training. Furthermore, the results of the present study show that the frequency of quarrels in families can be reduced by the training, at least in the judgment of mothers and especially for adolescents who play computer games, and that the adolescents have a higher willingness to discuss problem behaviour with their parents after the training. At the same time, the data show that parents’ irritability regarding children’s media use behaviour can be reduced through the ISES! Group Training. It is possible that the content regarding stress and relaxation for parents may have influenced this reduction. Overall, the ISES! Group Training was able to reduce conflicts in the families, increase communication about the problem behaviour, and reduce parents’ irritability.

### 4.3. Parents’ Burden

A total of 60% of the parents stated that they felt frequently and 65% severely burdened by their child’s media use. Furthermore, the parents of pathological or at-risk gamers were more stressed in the DASS than the parents of non- or inconspicuous gamers. The stress levels of the parents of at-risk and pathological gamers did not differ. This indicates that parents are as stressed by pre-clinical symptomatology as they are by clinical symptomatology. These findings are consistent with descriptions of high levels of distress among parents of adolescents with GD [21]. 

At the same time, the scores on the DASS indicated that, on average, the sample was not clinically distressed. This result expands on the heterogeneous findings to date that both have [23] and have not found [22] associations between depressive symptomatology in parents and IUD or GD symptomatology in children, while other studies have [22] or have not found [23] associations with anxiety.

Fathers showed higher depression scores after the intervention. It could be that, in terms of problem updating, the fathers are confronting issues they had previously avoided and are more concerned about the situation. Nevertheless, the depression scores before and after the training of the IG remained in the non-clinical range, so the significance of this result is mitigated.

### 4.4. Strengths, Limitations, and Perspectives

The strengths of this study lie in the study design (multicentre, randomized, controlled), the low dropout rate of the IG (0%), the high rate of participating fathers (39%), and the separate evaluation of mothers and fathers. Its limitations include the small sample size (especially for the subgroup analyses), the dominance of male adolescents (88%), and the high education level of the parents (88% college degree). The results are therefore not fully applicable to female adolescents and to less educated population groups. Another limitation is that 39% of the families already have or had contact with help. The survey did not allow a detailed evaluation of the type, frequency, and intensity of parallel interventions, so the results may be confounded by other interventions. Twenty-four percent of adolescents showed no conspicuous computer gaming behaviour in the parents’ judgment. Since no questionnaire on social network use was employed, it is not possible to conclusively assess whether this group represents adolescents with SNUD. Furthermore, most of the questionnaires (CSAS-FE, CIUS-P, EKI, PRSQ-P) were not validated in German. The conclusions are also limited because the data used are based on the parents’ subjective points of view and judgements. No objective measures for the adolescents were used, so improvements may also be explained by a change in the parents’ perceptions rather than a change in the situation. Further limitations so far, which can be overcome by the pending evaluations, are the missing follow-up data (6 and 12 months after the training) and a comparison with the questionnaire data of the studied adolescents. From a broader perspective, it would be desirable to combine the training with treatment for adolescents and to investigate whether the ISES! Group Training can further improve treatment outcomes. Furthermore, it would be interesting to conduct the training with families whose adolescents belong to a high-risk group for the development of an IUD (e.g., adolescents with obesity) [48].

## 5. Conclusions

- The ISES! Group training can decrease IUD symptomatology and GD symptomatology in at-risk users and increase adolescents’ readiness to change.
- Whether treatment readiness improves cannot be assessed based on the available data.
- The ISES! Group Training may enhance mother-child communication, increase the quality of the mother-child relationship, reduce family conflict, and possibly prevent escalation.
- The parents in this sample were not clinically distressed. Fathers showed higher sub-clinical depression scores after the intervention.

## Figures and Tables

**Table 1:** Contents of the ISES! Group Training (“Internet Addiction: Empowering Parents!”/original: “Internetsucht: Eltern staerken!”).

Session 1: Introduction and Psychoeducation
Detailed introduction of the participants. Information about addiction criteria, average Internet usage times in Germany, vicious circle model, influencing factors and common comorbidities. Exercise for home: define and write down a goal for the training.
Session 2: Parenting Behaviour and Parent-Child-Relationship
Information about positive, negative, short- and long-term consequences of the child’s behaviour. Development of ideas for exerting influence (e.g., acting as a role model regarding own leisure time activities and non-media emotion regulation, reducing co-dependent behaviour). Appreciation of the child’s competencies on the Internet. Information about the career aspirations “professional gamer” or “influencer”. Exercise for home: 1: Work on implementing the recommendations for consequences in handout (e.g., use timely, natural, positive, predictable, and reliable consequences). Home exercise 2 (due by session 4): Learn about child’s favourite Internet application in an appreciative way through dialogue with the child.
Session 3: Parenting Behaviour and Communication
Targeted use of positive or negative consequences (practical implementation of the theory developed). Reducing conflicts by getting to know the different sides of a message (“communication square”).
Session 4: Communication
Express needs and feelings (method of “non-violent communication”). Non-verbal communication (acting confident instead of aggressive or insecure). Exercise for home: try out non-violent communication
Session 5: De-escalation and Stress and Relaxation for Parents
Working out a typical escalation spiral and exit options using the strategies learned so far (e.g., communication, consequences). Dealing with aggressive behaviour and suicidal statements. Exercise for home: work through handout on stress and relief for parents (e.g., acute and chronic stress, ideas for stress reduction, breathing exercise instructions, relevance of role modelling for children’s stress regulation).
Session 6: Parent-Child Relationships
Strengthen parent-child relationships through positive time together. Encourage expressions of appreciation and positive affection. Promote alternative leisure activities for the child. Role model function regarding diverse and active leisure time activities by parents.

**Table 2:** Sample description.

Sociodemographic Data	Total Sample	IG	CG
**Parents**	N (%)	N (%)	N (%)
Mothers	36 (61%)	19 (57.5%)	17 (65.3%)
Fathers	23 (39.0%)	14 (42.4%)	9 (34.6%)
Participation with the partner	30 (50.9%)	18 (54.6%)	12 (46.2%)
People who feel they belong to the European culture	55 (93.2%)	32 (97.0%)	23 (88.5%)
School-leaving qualification: technical college or university entrance qualification	52 (88.1%)	28 (84.8%)	24 (92.3%)
**Children**			
Type of school: * German High School German Secondary School Other (e.g., community school)	24 (54.5%) 5 (11.4%) 12 (27.3%)	14 (58.3%) 2 (8.3%) 6 (25.0%)	10 (50.0%) 3 (15.0%) 6 (30.0%)
School attendance: * Regular Irregular Refusal	32 (72.7%) 5 (11.4%) 4 (9.1%)	17 (70.8%) 2 (8.3%) 3 (12.5%)	15 (75.0%) 3 (15.0%) 1 (5.0%)
Other people with media problems in the household: No Siblings Parent himself Partner Others	39 (66.1%) 13 (22.0%) 5 (8.5%) 2 (3.4%) 0 (0.0%)	18 (54.5%) 11 (33.3%) 4 (12.1%) 0 (0%) 0 (0.0%)	21 (80.8) 2 (7.7%) 1 (3.8%) 2 (7.7%) 0 (0.0%)
Actual regulation of media use: Not regulated Little regulated Moderately regulated Highly regulated	10 (16.9%) 19 (32.2%) 23 (39.0%) 7 (11.9%)	6 (18.2%) 6 (18.2%) 14 (42.4%) 7 (21.2%)	4 (15.4%) 13 (50.0%) 9 (34.6%) 0 (0%)
Type of media use of the child: Gaming Social media (incl. YouTube) Both	29 (49.2%) 14 (23.7%) 10 (16.9%)	15 (45.5%) 7 (21.2%) 8 (24.2%)	14 (53.8%) 7 (26.9%) 2 (7.7%)

Note: IG = intervention group; CG = control group; * The data of two parents regarding the same child were averaged.

**Table 3:** Results of adolescents’ symptomatology and their readiness for treatment (parents’ view) before and after the ISES! Group Training or the waiting period.

Scales		Pre	Post	Time × Group		
		*M (SD)*	*M (SD)*	$\eta_{p}^{2}$	F	*p*
CIUS-P total score	IG (N = 30)	41.57 (7.96)	38.40 (7.98)	0.08	4.22	0.045 *
	CG (N = 24)	40.50 (6.57)	40.54 (6.06)			
CSAS-FE total score	IG (N = 29)	36.79 (9.06)	33.28 (11.85)	0.018	0.92	0.342
	CG (N = 23)	33.91(8.95)	32.57 (10.36)			
CSAS-FE Subgroup: 0–1 criteria	IG (N = 7)	27.14 (5.08)	26.14 (8.92)	0.01	0.05	0.823
	CG (N = 5)	21.80 (3.27)	20.20 (2.17)			
CSAS-FE Subgroup: 2–4 criteria	IG (N = 9)	33.00 (3.67)	25.56 (3.84)	0.39	10.33	0.005 *
	CG (N = 9)	32.00(5.29)	33.89 (8.13)			
CSAS-FE Subgroup: ≥5 criteria	IG (N = 13)	44.62 (6.19)	42.46 (10.60)	0.01	0.27	0.611
	CG (N = 9)	42.56 (10.60)	38.11 (9.73)			
Single question: motivation to talk	IG (N = 33)	3.00 (2.18)	3.33 (2.50)	0.08	4.83	0.032 *
	CG (N = 26)	3.92 (2.61)	2.85 (1.98)			
Single question: motivation to change	IG (N = 33)	2.94 (1.95)	3.39 (2.46)	0.08	4.68	0.035 *
	CG (N = 26)	3.58 (2.42)	2.77 (1.61)			
Single question: motivation to seek help	IG (N = 33)	2.48 (1.99)	3.12 (2.91)	0.03	1.97	0.166
	CG (N = 26)	4.15 (3.04)	3.96 (3.23)			

Note: IG = intervention group; CG = control group; CIUS-P = Compulsive Internet Use Scale—Parental Version; CSAS-FE = Computer Game Addiction Scale—Parents Version; * *p* ≤ 0.05.

**Table 4:** Significant and trending results of parent-child relationship before and after ISES! Group Training or the waiting period.

Scales		Pre	Post	Time × Group		
		*M (SD)*	*M (SD)*	$\eta_{p}^{2}$	F	*p*
EKI child empathy—parents’ view	IG (N = 32)	2.32 (0.88)	2.63 (0.93)	0.06	3.29	0.075
	CG (N = 26)	2.37 (1.10)	2.38 (0.83)			
EKI child empathy—mother’s view	IG (N = 19)	2.30 (0.91)	2.72 (1.02)	0.09	3.49	0.070
	CG (N = 17)	2.37 (1.11)	2.39 (0.84)			
EKI child authenticity—mother’s view	IG (N = 19)	2.84 (0.94)	3.11 (0.99)	0.14	5.48	0.025 *
	CG (N = 17)	2.90 (1.04)	2.71 (1.04)			
PRSQ-P cohesion—mother’s view	IG (N = 19)	2.99 (0.54)	3.06 (0.59)	0.08	2.94	0.096
	CG (N = 17)	3.08 (0.50)	2.93 (0.50)			
PRSQ-P aid—mother’s view	IG (N = 19)	1.11 (0.50)	1.18 (0.55)	0.09	3.28	0.079
	CG (N = 16)	0.94 (0.51)	0.72 (0.44)			
PRSQ-P emotional burden—parents’ view	IG (N = 32)	1.34 (0.50)	1.46 (0.54)	0.10	5.90	0.018 *
	CG (N = 25)	1.08 (0.50)	0.95 (0.58)			
PRSQ-P emotional burden—mother’s view	IG (N = 18)	1.47 (0.46)	1.65 (0.56)	0.17	6.71	0.014 *
	CG (N = 16)	1.23 (0.52)	1.08 (0.64)			

Note: IG = intervention group; CG = control group; EKI = Parent-Child Inventory; PRSQ-P = Parental Representation Screening Questionnaire—Parental Version; * *p* ≤ 0.05.

**Table 5:** Results of parent’s burden before and after ISES! Group Training or the waiting period.

		Pre	Post	Time × Group		
		*M (SD)*	*M (SD)*	$\eta_{p}^{2}$	F	*p*
**DASS: total value**						
Both parents	IG (N = 27)	13.0 (9.10)	13.85 (11.14)	0.033	1.63	0.207
	CG (N = 23)	9.65 (6.46)	7.91 (6.32)			
Mothers	IG (N = 15)	13.53 (9.34)	12.53 (7.70)	0.00	0.03	0.874
	CG (N = 15)	9.27 (6.19)	8.67 (6.77)			
Fathers	IG (N = 12)	12.33 (9.15)	15.50 (14.58)	0.20	4.44	0.049 *
	CG (N = 8)	10.38 (7.31)	6.50 (5.53)			
**DASS: subscale depression**						
Both parents	IG (N = 31)	4.61 (4.67)	5.48 (5.71)	0.06	3.33	0.073
	CG (N = 24)	3.71 (3.47)	2.54 (3.32)			
Mothers	IG (N = 17)	4.41 (4.85)	4.47 (4.78)	0.01	0.35	0.558
	CG (N = 16)	3.69 (2.87)	2.82 (2.99)			
Fathers	IG (N = 14)	4.86 (4.61)	6.71 (6.64)	0.23	5.83	0.025 *
	CG (N = 8)	3.75 (4.68)	2.00 (4.07)			
**DASS: subscale anxiety**						
Both parents	IG (N = 31)	2.00 (2.07)	2.16 (2.99)	0.00	0.01	0.909
	CG (N = 24)	1.04 (1.40)	1.13 (1.48)			
Mothers	IG (N = 18)	2.50 (2.38)	1.56 (1.65)	0.07	2.29	0.140
	CG (N = 15)	0.93 (1.49)	1.07 (1.22)			
Fathers	IG (N = 13)	1.31 (1.32)	3.00 (4.14)	0.09	2.02	0.171
	CG (N = 9)	1.22 (1.30)	1.22 (1.92)			
**DASS: subscale stress**						
Both parents	IG (N = 33)	8.79 (5.35)	8.67 (5.25)	0.001	0.06	0.800
	CG (N = 26)	6.23 (4.35)	5.85 (4.71)			
Mothers	IG (N = 19)	9.05 (5.40)	8.42 (4.51)	0.01	0.231	0.634
	CG (N = 17)	6.12 (4.69)	6.18 (4.85)			
Fathers	IG (N = 14)	8.43 (5.46)	9.00 (6.28)	0.06	1.41	0.249
	CG (N = 9)	6.44 (3.88)	5.22 (4.63)			

Note: IG = intervention group; CG = control group; DASS: Depression, Anxiety, and Stress Scale; Cut-off for subscale depression: 10; Cut-off for subscale anxiety: 6; Cut-off for subscale stress: 10; * *p* ≤ 0.05.

## Data Availability

Access to data may be provided upon request.

## Supplementary Materials

The following supporting information can be downloaded at: https://www.mdpi.com/article/10.3390/ijerph20010272/s1, Table S1: All results of parent-child relationship before and after ISES! Group Training or the waiting period.
