# Supplementary material for: Randomized Controlled Evaluation of a Group-Based Training for Parents of Adolescents with Gaming Disorder or Social Network Use Disorder
Source: Int J Environ Res Public Health. 2022 Dec 24;20(1):272. doi: 10.3390/ijerph20010272 (PMC9819552; doi:10.3390/ijerph20010272)
Supplement: Supplementary file 1 [file ijerph-20-00272-s001.zip › ijerph-2036141-supplementary.pdf]

Supplementary Table S1: All results of parent-child relationship before and after ISES! Group Training or the waiting period.

Scales		pre	post	time x group		
		M (SD)	*M (SD)*	η_p^2	F	*p*
PRSQ-P cohesion – parents' view	IG (N=33)	2.95 (0.56)	2.98 (0.58)	0.01	0.71	0.400
	CG (N= 26)	2.88 (0.63)	2.82 (0.55)			
PRSQ-P cohesion – father's view	IG (N= 14)	2.89 (0.59)	2.86 (0.57)	0.06	1.26	0.274
	CG (N=9)	2.49 (0.71)	2.62 (0.63)			
PRSQ-P autonomy – parents' view	IG (N=32)	2.52 (0.47)	2.41 (0.52)	0.03	1.68	0.200
	CG (N=25)	2.59 (0.41)	2.60 (0.45)			
PRSQ-P autonomy – mother's view	IG (N=19)	2.45 (0.49)	2.33 (0.53)	0.02	0.56	0.460
	CG (N=16)	2.61 (0.43)	2.58 (0.51)			
PRSQ-P autonomy – father's view	IG (N=13)	2.62 (0.43)	2.52 (0.52)	0.063	1.34	0.063
	CG (N=9)	2.56 (0.39)	2.64 (0.33)			
PRSQ-P resources - parents' view	IG (N=33)	2.71 (0.39)	2.67 (0.41)	0.00	0.02	0.890
	CG (N=26)	2.72 (0.41)	2.69 (0.39)			
PRSQ-P resources – father's view	IG (N=14)	2.70 (0.40)	2.63 (0.40)	0.17	4.21	0.053
	CG (N=9)	2.52 (0.40)	2.63 (0.2.6)			
PRSQ-P resources – mother view	IG (N=19)	2.72 (0.39)	2.70 (0.37)	0.02	0.71	0.405
	CG (N=17)	2.82 (0.38)	2.71 (0.45)			
PRSQ-P conflicts – parents' view	IG (N=33)	2.36 (0.57)	2.30 (0.55)	0.04	2.44	0.120
	CG (N=25)	2.15 (0.55)	2.22 (0.65)			
PRSQ-P conflicts – mother's view	IG (N=19)	2.37 (0.653)	2.32 (0.59)	0.02	0.51	0.479
	CG (N=16)	2.19 (0.57)	2.22 (0.66)			
PRSQ-P conflicts – father's view	IG (N=14)	2.36 (0.46)	2.27 (0.51)	0.11	2.70	0.115
	CG (N=9)	2.08 (0.53)	2.22 (0.67)			
PRSQ-P punishment – parents' view	IG (N=32)	0.34 (0.35)	0.36 (0.32)	0.00	0.01	0.940
	CG (N=26)	0.28 (0.31)	0.31 (0.34)			
PRSQ-P punishment – mother's view	IG (N=19)	0.33 (0.35)	0.35 (0.34)	0.01	0.43	0.518
	CG (N=17)	0.33 (0.35)	0.29 (0.33)			
PRSQ-P punishment – father's view	IG (N=13)	0.36 (0.37)	0.38 (0.30)	0.06	1.30	0.270
	CG (N=9)	0.19 (0.18)	0.33 (0.37)			
PRSQ-P rejection - parents' view	IG (N=32)	0.27 (0.36)	0.27 (0.35)	0.01	0.30	0.590
	CG (N=25)	0.22 (0.28)	0.19 (0,28)			
PRSQ-P rejection - mother's view	IG (N=19)	0.30 (0.41)	0.26 (0.28)	0.01	0.19	0.668
	CG (N=17)	0.18 (0.23)	0.18 (0.26)			
PRSQ-P rejection - father's view	IG (N=13)	0.21 (0.29)	0.29 (0.44)	0.12	2.64	0.121
	CG (N=8)	0.31 (0.37)	0.22 (0.34)			
PRSQ-P fears/overprotection - parents' view	IG (N= 32)	1.77 (0.75)	1.96 (0.86)	0.03	1.89	0.170
	CG (N=26)	1.88 (0.80)	1.91 (0.92)			
PRSQ-P fears/overprotection - mother's view	IG (N=18)	1.83 (0.66)	2.07 (0.81)	0.02	0.02	0.442
	CG (N=17)	2.04 (0.87)	2.16 (0.90)			
PRSQ-P fears/overprotection - father's view	IG (N=14)	1.68 (0.87)	1.82 (0.94)	0.09	1.98	0.174
	CG (N=9)	1.58 (0.59)	1.44 (0.80)			
PRSQ-P aid - parents' view	IG (N=33)	1.11 (0.50)	1.11 (0.50)	0.04	2.17	0.150
	CG (N=25)	0.93 (0.45)	0.76 (0.42)			
PRSQ-P aid - father's view	IG (N=14)	1.11 (0.52)	1.02 (0.41)	0.00	0.00	0.97
	CG (N=9)	0.92 (0.35)	0.83 (0.42)			
	IG (N=14)	1.16 (0.51)	1.21 (0.40)	0.03	0.57	0.458

PRSQ-P emotional burden - father's view	CG (N=9)	0.81 (0.33)	0.72 (0.40)			
PRSQ-P risk - parents' view	IG (N=33)	1.24 (0.32)	1.30 (0.31)	0.03	1.55	0.220
	CG (N=26)	1.15 (0.31)	1.14 (0.37)			
PRSQ-P risk - mother's view	IG (N=19)	1.29 (0.31)	1.34 (0.26)	0.02	0.77	0.387
	CG (N=17)	1.22 (0.33)	1.21 (0.37)			
PRSQ-P risk - father's view	IG (N=14)	1.17 (0.33)	1.23 (0.36)	0.04	0.76	0.390
	CG (N=9)	1.02 (0.25)	1.02 (0.34)			
PRSQ-P total value - parents' view	IG (N=28)	1.54 (0.51)	1.46 (0.50)	0.01	0.17	0.690
	CG (N=22)	1.69 (0.46)	1.66 (0.54)			
PRSQ-P total value - mother's view	IG (N=17)	1.40 (0.60)	1.31 (0.52)	0.00	0.00	1.000
	CG (N=14)	1.74 (0.46)	1.65 (0.64)			
PRSQ-P total value - father's view	IG (N=11)	1.74 (0.26)	1.70 (0.36)	0.06	1.16	0.297
	CG (N=8)	1.61 (0.48)	1.69 (0.32)			

Note: IG = intervention group; CG = control group; EKI = Parent-Child Inventory; PRSQ-P = Parental Representation Screening Questionnaire – Parental Version
